# London's Hospital Sunday Yields £85,000

**Published:** 1918-08-17

**Authors:** 


### LONDON'S HOSPITAL SUNDAY YIELDS £85,000.

For decades of years the Metropolitan press backed public men of every view who generously gave their aid by important speeches to awaken the inhabitants of the Metropolis to the claims of the voluntary hospitals and the importance of the Hospital Sunday Fund movement. The table of figures given on page 14 of the Metropolitan Hospital Sunday Fund Report for the year ending October 31, 1917, exhibits the rise and fall in the amounts received from congregations extending over a period of forty-five to forty-six years. These figures are full of interest and should be studied.

The fourth year of the Great War has kept the newspapers full of war news, and every able-bodied person, both man and woman, to a greater or less extent wholly or over occupied with the business of the war. It was not, therefore, a favourable year on the face of it to increase to an adequate sum the congregational collections and other sources of revenue on which Hospital Sunday in London relies for its existence and success. In fact, the zealous advocates of the Sunday Fund claim that the able speakers usually backing it were all previously engaged, and over-full with other pressing affairs, so that Hospital Sunday, 1918, had to be left entirely to the consciences and generosity of attendants at places of worship and the ordinary casual channels of supply. Nothing daunted, Sir Ernest Tritton, the able and indefatigable Vice-Chairman of the Fund, and the untiring, continuous, and successful work of the office staff, led by the Secretary, Mr. Arnold James, produced the best organised effort for increasing the amount received from congregational collections ever attempted during the forty-seven years of the Fund's existence. These efforts were nobly supported by the clergy. We have authority for stating that 85 per cent. of the 1,100 church collections received by the 8th inst. show an increase over their contributions in previous years. No more striking sign of the vitality of the Metropolitan Hospital Sunday Fund could surely be asked for, and it reflects the greatest credit upon the ministers and all workers who cooperated with them to attain these results, which in the aggregate have yielded a total collection so large as to exceed by £15,000 the yield of the previous best year in the Fund's history.

The clergy may be warmly congratulated upon these results, which have been attained, it must be remembered, at a time when upwards of seven millions of Britons are away on war service fighting for the freedom, protection, and defence of their families and their homes. The list of contributing congregations exhibits, however, some remarkable achievements. Never before has a single congregation given on any Hospital Sunday £2,453, the amount collected at St. Mark's, North Audley Street. Kensington Parish Church, under the new vicar, by the adoption of the house-to-house collection method, contributed £441, against £278 in the previous year, whilst St. Michael's, Cornhill, raised £121 this year against £6 formerly. We have not space to give all the results, but cannot omit to mention St. Andrew's, Lambeth, a very poor district indeed, which has this year raised the remarkable sum of £76, the vicar being a zealous and devoted worker. London owes much to its churches, clergy, ministers, and citizens. The voluntary hospitals owe a debt of acknowledgment and thanks to Sir Ernest Tritton, who accepted the office of Vice-Chairman when the Fund was under a cloud in 1914, and has devoted himself to its interests and development with self-denial, producing the splendid results it has been our grateful privilege to set forth in this article.

